# Invasive urodynamic testing prior to surgical treatment for stress urinary incontinence in women: cost-effectiveness and value of information analyses in the context of a mixed methods feasibility study

**DOI:** 10.1186/s40814-018-0255-y

**Published:** 2018-03-23

**Authors:** Tara Homer, Jing Shen, Luke Vale, Elaine McColl, Douglas G. Tincello, Paul Hilton, on behalf of the INVESTIGATE-I studies group, Natalie Armstrong, Catherine Brennand, Denise Howel, Andrew Bryant, Malcolm Lucas, Brian Buckley, Christopher Chapple

**Affiliations:** 1 Health Economics Group, Institute of Health & Society, Newcastle University, Baddiley-Clark Building, Richardson Road, Newcastle upon Tyne, NE2 4AX UK; 2 Institute of Health and Society, Newcastle University, Newcastle upon Tyne, UK; 3 Department of Health Sciences, University of Leicester, Leicester, UK; 4 Faculty of Medical Sciences, Newcastle University, Newcastle upon Tyne, UK

**Keywords:** Randomised controlled pilot trial, Feasibility study, Cost-effectiveness, Value of information, Invasive urodynamic testing, Stress urinary incontinence

## Abstract

**Background:**

INVESTIGATE-I (INVasive Evaluation before Surgical Treatment of Incontinence Gives Added Therapeutic Effect?) was a mixed methods study to assess the feasibility of a future randomised controlled trial of invasive urodynamic testing (IUT) prior to surgery for stress urinary incontinence (SUI) in women. Here we report one of the study’s five components, with the specific objectives of (i) exploring the cost-effectiveness of IUT compared with clinical assessment plus non-invasive tests (henceforth described as ‘IUT’ and ‘no IUT’ respectively) in women with SUI or stress-predominant mixed urinary incontinence (MUI) prior to surgery, and (ii) determining the expected net gain (ENG) from additional research.

**Methods:**

Study participants were women with SUI or stress-predominant MUI who had failed to respond to conservative treatments recruited from seven UK urogynaecology and female urology units. They were randomised to receive either ‘IUT’ or ‘no IUT’ before undergoing further treatment. Data from 218 women were used in the economic analysis. Cost utility, net benefit and value of information (VoI) analyses were performed within a randomised controlled pilot trial. Costs and quality-adjusted life years (QALYs) were estimated over 6 months to determine the incremental cost per QALY of ‘IUT’ compared to ‘no IUT’. Net monetary benefit informed the VoI analysis. The VoI estimated the ENG and optimal sample size for a future definitive trial.

**Results:**

At 6 months, the mean difference in total average cost was £138 (*p* = 0.071) in favour of ‘IUT’; there was no difference in QALYs estimated from the SF-12 (difference 0.004; *p* = 0.425) and EQ-5D-3L (difference − 0.004; *p* = 0.725); therefore, the probability of IUT being cost-effective remains uncertain. The estimated ENG was positive for further research to address this uncertainty with an optimal sample size of 404 women.

**Conclusions:**

This is the largest economic evaluation of IUT. On average, up to 6 months after treatment, ‘IUT’ may be cost-saving compared to ‘no IUT’ because of the reduction in surgery following invasive investigation. However, uncertainty remains over the probability of ‘IUT’ being considered cost-effective, especially in the longer term. The VoI analysis indicated that further research would be of value.

**Trial registration:**

ISRCTN. ISRCTN71327395. Registered 7 June 2010.

**Electronic supplementary material:**

The online version of this article (10.1186/s40814-018-0255-y) contains supplementary material, which is available to authorized users.

## Background

Urinary incontinence (UI) affects 25–45% of women aged 15 years and older [1]. The most prevalent types are stress urinary incontinence (SUI) and stress-predominant mixed urinary incontinence (MUI), which jointly account for 65–85% of cases [2].

Current National Institute for Health and Care Excellence (NICE) guidelines state that cystometry is not required prior to conservative treatments or surgery when a diagnosis of SUI is likely on clinical grounds (i.e. where there are no symptoms of over active bladder or voiding dysfunction, no anterior compartment prolapse, and no previous surgery for SUI) [3, 4].

A number of previous studies have considered the use of invasive urodynamic testing (IUT) in determining the optimum treatment strategy for SUI [5–8]. However, differences in inclusion criteria and methods used mean that there is continuing uncertainty over the current position of IUT in the diagnostic pathway of SUI [9, 10]. IUT use in the UK varies; a 2002 survey of gynaecology and urology units performing IUT found that only half had a guideline on the indications for its use and 85% performed cystometry, the most commonly used form of IUT, in all women with UI [11].

The continuing uncertainty over the clinical utility of urodynamic investigation resulted in calls for further high-quality primary research from major UK and international bodies [12–15]. As a result, the UK National Institute for Health Research – Health Technology Assessment programme (NIHR-HTA) commissioned a mixed methods feasibility study (INVESTIGATE-I), including an external pilot randomised controlled trial, in order to clarify how best to address this uncertainty [16]. Given the limited evidence on the cost-effectiveness of IUT [15], an economic evaluation was incorporated in the INVESTIGATE-I study. This study is the first to concurrently estimate the clinical and cost-effectiveness of IUT in this population [5, 8]. The economic evaluation included cost utility, net monetary benefit (NMB) and value of information (VoI) analyses. The aims of the economic components, reported here, were to inform an economic analysis for a future definitive clinical trial and to estimate the sample size required to achieve a definitive answer on the cost-effectiveness of IUT.

## Methods

The INVESTIGATE-I randomised controlled pilot trial was undertaken in seven centres across the UK. Full details of inclusion and exclusion citeria, other aspects of the study design, and clinical effectiveness outcomes are reported elsewhere [16–18].

Participants randomised within INVESTIGATE-I had a clinical diagnosis of SUI or stress-predominant MUI and were about to undergo surgical treatment. The aim of the study was to evaluate whether IUT, compared with basic clinical assessment and non-invasive tests, altered treatment decisions and outcomes in women suitable for surgical treatment. The treatments provided as part of the study included surgical and non-surgical treatments. Participants had a 6 month follow-up period and both costs and utilities were the primary outcomes for the economic evaluation. Treatment costs, including information on IUT, were collected via a case report form (CRF) completed by clinical staff during the woman’s initial treatment phase. Follow-up resource use was collected via a self-completed Participant Costs Questionnaire completed at 6 months. Data on utilities were based on responses to the self-completed SF-12 and EQ-5D-3L administered at baseline and 6 months. Further information on the estimation of costs and utilities is provided under “Estimation of costs” and “Estimation of effects”.

Between April 2011 and December 2012, 771 women were assessed for eligibility, and of these, 110 were randomised to the control arm (‘no IUT’) and 112 to the intervention arm (‘IUT’). The economic evaluation was conducted from the perspective of the UK National Health Service (NHS).

### Estimation of costs

Healthcare resource use and costs were estimated for all women using data collected within the trial and from routine sources [19, 20]. Resource use and costs were categorised as those used prior to treatment, to determine the optimum treatment strategy (i.e. the costs of IUT), those used in providing treatment (i.e. the costs of surgery and alternative non-surgical treatments), and those incurred after treatment (i.e. other healthcare resources used over a 6 month period following the start of treatment). We assumed non-invasive investigations would be similar across both arms and hence excluded these from our analysis. There may be an argument for reporting the investigation costs prior to treatment separately from those incurred by and after treatment. However, since the former are of necessity different (favouring ‘no IUT’), it is our view that the cost of the total package of care (before, during and after treatment) is the more relevant outcome.

#### Resources and costs prior to treatment

The cost of IUT was estimated using a micro-costing approach [21], which provides a more accurate estimation of costs than a ‘top-down’ approach [21, 22]. Data on resource use of the investigation are presented in Additional file 1, so that readers can judge the applicability to their own circumstances. Although conventional dual-channel subtracted cystometry with simultaneous pressure/flow voiding studies (henceforth abbreviated to ‘*cystometry*’), videourodynamics and ambulatory urodynamics were all permitted within the study protocol, only cystometry was costed as it was performed in 92% of women randomised to ‘IUT’. Thus, if any IUT was undertaken, the cost of cystometry was used. The consumable resources used in cystometry were obtained from clinical staff at one participating site (Liz Dixon, personal communication, July 2013). The cost of the primary staff member who performed the investigation was based on information, collected via a CRF, on the length of time in the consulting room and staff grade. Based on clinical advice, we judged that a nurse (UK band 5) would also be present for the IUT, so this cost was included in the ‘IUT’ cost. Salary costs were obtained from routine sources [20].

#### Resources and costs required to provide treatment

The expected treatment for those randomised to ‘no IUT’ was surgery. The initial treatment for women randomised to ‘IUT’ was based on the findings from investigation and could include surgical and non-surgical approaches. The cost of surgery was taken from the NHS reference cost [19] for ‘vaginal tape operations for urinary incontinence’. All non-surgical treatments: behaviour modification, bladder retraining, and pelvic floor muscle training, were micro-costed based on personal communication and NHS guidelines [3] with further details provided in Additional file 1. Five women deferred initial treatment and were classified as ‘watchful waiting’; it was assumed they would still use containment products provided by the NHS. This cost was obtained from a recent HTA on non-surgical management of SUI, [23] inflated to 2015 prices [24].

#### Resource use and costs of services used after treatment

The Patient Costs Questionnaire, completed 6 months after surgery or the start of non-surgical treatment, asked about women’s use of primary and secondary care services following initial treatment. Primary care costs included visits to the general practitioner (GP), practice nurse, continence nurse, community physiotherapist and prescriptions. Costs of prescription were estimated from a routine source [20] assuming a standard GP prescription charge. Secondary care included inpatient and outpatient visits and their costs also came from a routine source [19].

### Estimation of effects

Quality-adjusted life years (QALYs) were estimated using the area under the curve approach [25] for every woman, based on responses to the SF-12 and EQ-5D-3L completed at baseline and 6 months. Both the SF-12 and the EQ-5D-3L are widely used in the measurement of health-related quality of life and are applicable to a wide range of health conditions and treatments [26, 27]. Multiple imputation [21] was used to estimate missing QALY values, controlling for randomised allocation and age. We assumed missing data were missing at random [22].

### Economic evaluation

The cost-utility analysis used an intention-to-treat principle. Results were presented as point estimates of the mean incremental costs, QALYs and cost per QALY, estimated using both the trial data (unadjusted analysis) and seemingly unrelated regression (SUR) [28]. The SUR analysis is considered to be more reliable as it improves precision surrounding cost-effectiveness estimates [29]. SUR permits the simultaneous estimation of costs and QALYs, calculated at an individual level, while accounting for unobserved individual characteristics that could affect both costs and QALYs and lead to potential correlation between these two variables [29]. Both equations controlled for randomised allocation to estimate the difference in costs and QALYs between the two arms. The QALY equation also controlled for baseline utility and age as these can cause an imbalance in the QALY estimate [30]. An intervention is typically deemed cost-effective when the incremental cost-effectiveness ratio (ICER) is less than £20,000 per QALY gained [31].

### Sensitivity analysis

A stochastic sensitivity analysis, using the bootstrapping technique [32], explored the impact of the statistical imprecision surrounding estimates of costs, effects and cost-effectiveness. Uncertainty surrounding the cost-effectiveness ratio was presented on the cost-effectiveness plane [33]. Confidence intervals and *p* values were estimated by the SUR model to identify any statistically significant difference in costs and effects. Other uncertainties addressed using sensitivity analyses are presented elsewhere [16]. Due to the short follow-up period, costs and QALYs were not discounted.

### Net monetary benefit

The NMB was calculated at the widely used UK willingness-to-pay (WTP) threshold of £20,000 [31]. The WTP threshold is the opportunity cost of reallocating resources for one additional QALY. This allows conversion of health benefits (QALYs) into a common metric of pounds sterling by multiplying QALYs by WTP (see equation below). NMB allows us to make comparisons among multiple treatments as well as enabling the VoI analysis. Confidence intervals were estimated from the bootstrapped results.

$$ \mathrm{NMB}=\left[{\lambda}^{\ast}\Delta E-\Delta C\right] $$

where *λ* is the willingness-to-pay threshold; *Δ* is the difference between the two randomised arms (‘IUT’ and ‘no IUT)’; *E* is the effects; and *C* is the costs.

### Value of information

The expected value of sampling information (EVSI) [34] method was used to predict the costs and benefits of obtaining further information based on current information. The EVSI model was estimated by imputing the trial data in to the mathematical derivations and equations presented by Willan and Pinto [34]. The principle underpinning EVSI is that the allocation of funds for research to reduce clinical and economic uncertainties has financial and opportunity costs. The financial cost is the total cost incurred from conducting further research. The opportunity cost is the delay in making and implementing recommendations on the use of IUT, caused by undertaking additional research.

EVSI determines the expected net gain (ENG) from future research, i.e. that the expected added value of further research minus the (financial and opportunity) costs of that research. A positive ENG indicates that the added value of additional research exceeds the costs. EVSI also enables the prediction of the economically and statistically efficient sample size in each randomised arm of a future economic evaluation as part of a randomised controlled trial. This sample size was then doubled to account for a two-arm trial and inflated to account for dropouts—in this case, the rate of non-response to the SF-12 and EQ-5D-3L questionnaires. The optimal sample size in this context is the total number of complete cases that would maximise the ENG and provides a definitive economic answer on the use of IUT.

The economic evaluation was conducted in Stata using the seemingly unrelated regression command *sureg* (StataCorp LLC, Texas, USA). The VoI analysis was performed using EXCEL and further details are available from the lead author on request.

## Results

There were 222 women initially randomised in INVESTIGATE-I. Information on 218 women was used in the economic analysis; 110 in the ‘IUT’ arm and 108 in the ‘no IUT’ arm. Four women were excluded due to the following: withdrawal (*n* = 2), ineligibility (*n* = 1) and an error in the participant’s unique identifier (*n* = 1).

### Costs

In terms of initial treatment, 74% of women underwent surgery in the ‘IUT’ arm compared to 94% of women in the ‘no IUT’ arm (Fisher’s exact *p* < 0.01). There were no other statistically significant differences in healthcare resources used during the follow-up period between the two arms (Additional file 2). There was a difference of £138 (95% CI − £288, £12; *p* = 0.071), in average total cost per woman between trial arms (Table 1), driven by the lower number of women undergoing surgical treatment in the ‘IUT’ arm. The difference in costs suggests that IUT has the potential to be cost-saving, although as a pilot study, our trial was not powered to achieve statistical significance on this or other outcomes.

**Table 1:** Cost-effectiveness results

Investigation strategy	Total avg. cost per woman (£)	Incremental cost (£)	Total avg. QALYs per woman	Incremental QALYs	ICER (£)	Probability that IUT is cost-effective for different threshold values for society’s willingness to pay for a QALY				
SF-12 results						£0 K	£10 K	£20 K	£30 K	£50 K
‘no IUT’	1489		0.377			0.04	0.03	0.05	0.09	0.12
‘IUT’	1351	− 138	0.385	0.008	Dominant	0.96	0.97	0.95	0.91	0.88
‘no IUT’ adjusted^a^						0.04	0.03	0.04	0.07	0.1
‘IUT’ adjusted^a^		− 138		0.004	Dominant	0.96	0.97	0.96	0.93	0.9
EQ-5D-3L results										
‘no IUT’	1489		0.413	0.018	7667	0.97	0.80	0.60	0.51	0.43
‘IUT’	1351	− 138	0.395			0.03	0.20	0.40	0.49	0.57
‘no IUT’ adjusted^a^				0.004	34,500	0.96	0.84	0.64	0.54	0.46
‘IUT’ adjusted^a^		− 138				0.04	0.16	0.36	0.46	0.54

^a^Results reported from SUR estimation; adjusting for randomisation in the cost equation and for randomisation, baseline utility (estimated from both the SF12 and EQ-5D-3L respectively), and age in the HRQoL equation

### Effects

QALY values could only be estimated for women who completed the SF-12 or EQ-5D-3L at both baseline and 6 months (Additional file 3). The response rates to both questionnaires were relatively low with QALYs being estimated for only half of the participants having both baseline and 6 month utility data. The uncertainty caused by the dropout rate was incorporated into the analyses. The uncertainty surrounding estimates of costs, effects and overall cost-effectiveness are presented in the cost-effectiveness plane. A complete case analysis was also performed and found similar results [16]. The SF-12 SUR results, presented in Table 1, slightly favoured ‘IUT’ (diff 0.004; 95% CI − 0.006, 0.013: *p* = 0.425) whereas the EQ-5D-3L SUR results slightly favoured ‘no IUT’ (diff − 0.004; 95% CI − 0.024, 0.016: *p* = 0.725). However, both differences were close to zero, and neither were statistically significant, indicating that there is no evidence of a difference in benefits in terms of QALYs.

### Economic evaluation

We estimated the ICER, using QALY estimates derived from both the SF-12 and EQ-5D-3L, using unadjusted and SUR analyses (Table 1). Using the SF-12 results, ‘IUT’ was the dominant investigation strategy as it was more effective and less costly (albeit the differences in costs and effects were not statistically significant). Based on the EQ-5D-3L results, ‘no IUT’ generated more benefits than ‘IUT’ but it was also more costly; hence, an ICER was derived in order to judge the preferred investigation strategy. The value of the ICER varied markedly between the unadjusted and SUR analyses, ‘no IUT’, with an ICER of £34,500, would not be considered cost-effective in the adjusted analysis at current WTP thresholds [31].

The economic evaluation results are useful for determining the balance of probabilities which can help to inform decision makers. The balance of probabilities is the probability of IUT being considered cost-effective at different WTP values (Table 1). Using the SF-12 results, IUT was the investigation strategy with the higher probability of being cost-effective at all WTP values up to and including £30,000 [31].

#### Sensitivity analysis

**Fig. 1:**
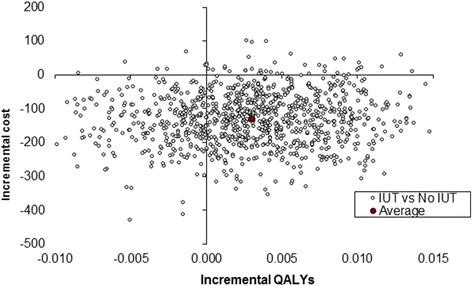
Incremental cost-utility scatterplot derived from the SF-12 data

**Fig. 2:**
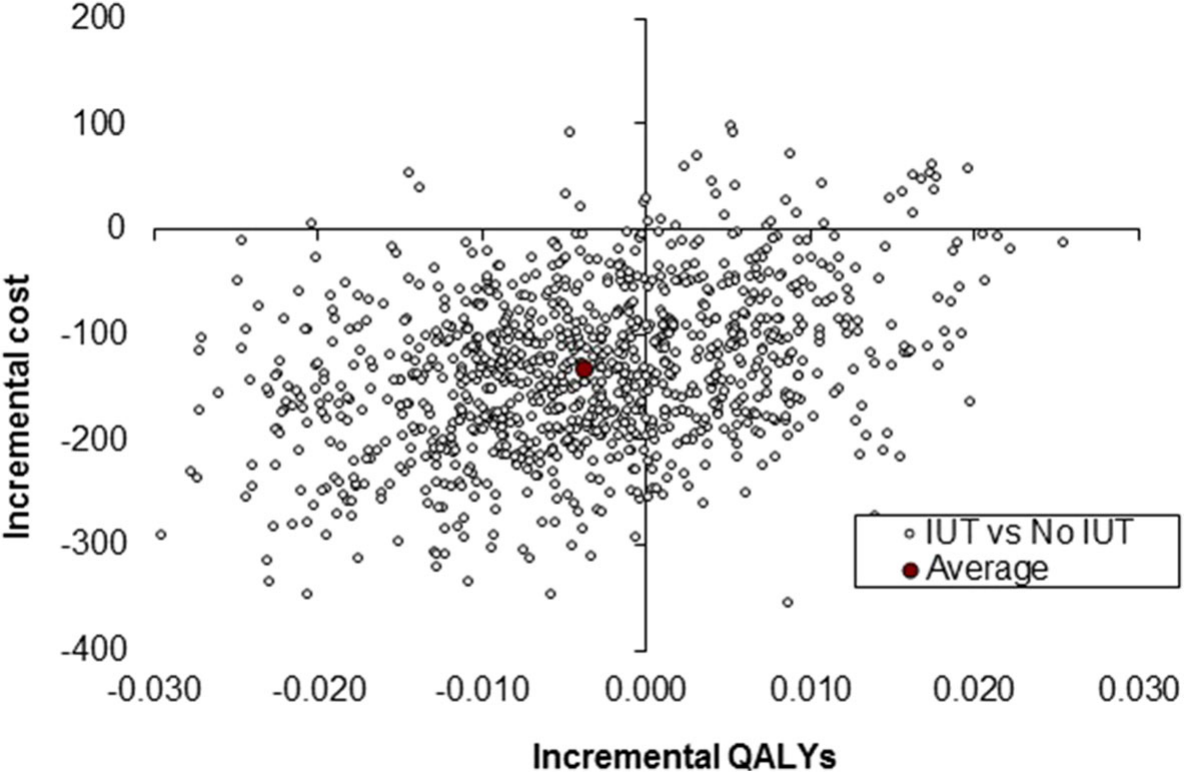
Incremental cost-utility scatterplot derived from the EQ-5D-3L data

Stochastic sensitivity analyses were performed to test the robustness of the results. The imprecision surrounding these estimates is shown on the cost-effectiveness plane (Figs. 1 and 2). The majority of iterations for both results were in the southern quadrants, supporting the suggestion of cost savings associated with IUT. In Fig. 1 (derived from the SF-12), the average of the bootstrapped iterations was in the south-east quadrant, highlighting that on average IUT was more effective in terms of both costs and QALYs. In Fig. 2 (derived from the EQ-5D-3L), the average of the bootstrapped iterations was positioned in the south-west quadrant suggesting that whilst IUT, on average, was less costly it was also less effective in terms of QALYs. In both figures, the average bootstrapped iteration lies close to the *y*-axis emphasising that there was no significant difference in effects.

### Net monetary benefit

We imputed the unadjusted SF-12 cost-effectiveness results into the NMB equation. The NMB was £160 (95% CI £144, £235) in favour of ‘IUT’. The unadjusted EQ-5D-3L cost-effectiveness results were used in a sensitivity analysis.

### Value of information analysis

**Fig. 3:**
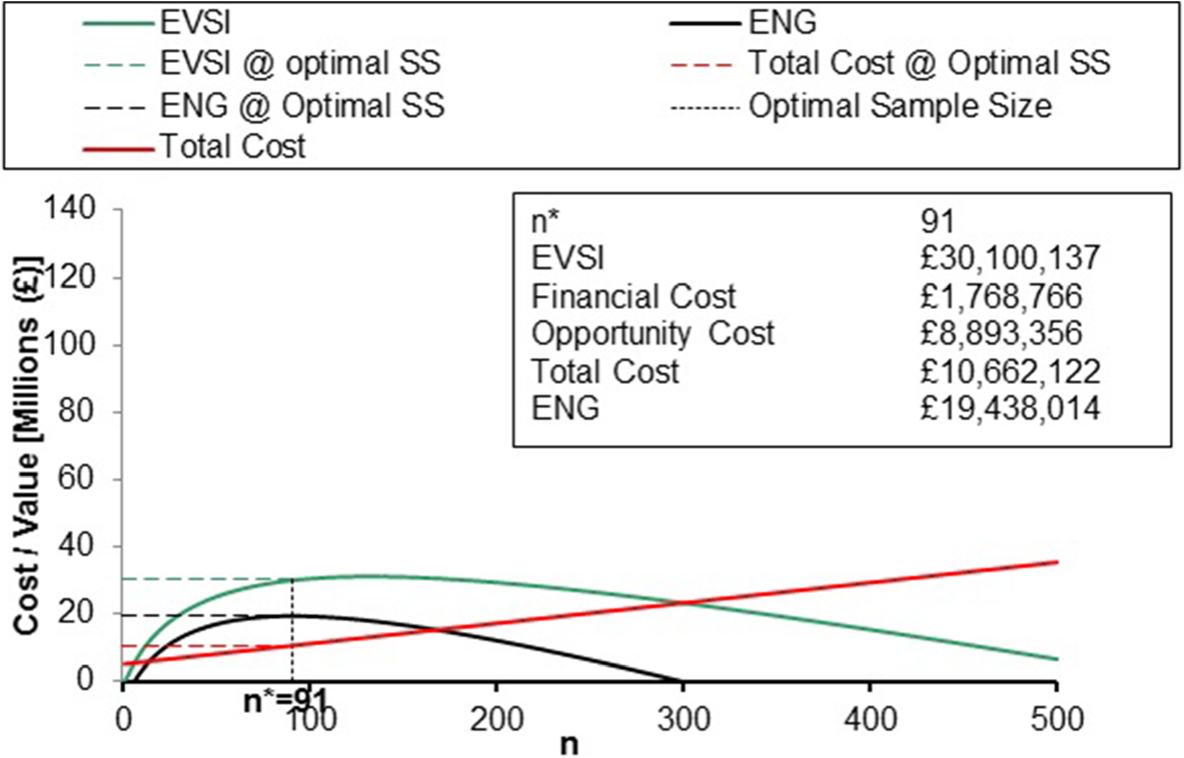
Expected value of sampling information derived from SF-12 data

The assumption for the EVSI, based on the NMB results, was to estimate the additional value of future research to determine whether ‘IUT’ was cost-effective compared to ‘no IUT’. The parameters used and the results of the EVSI model are presented in Additional file 4 and Fig. 3 respectively.

The results estimated from the EVSI model suggest there is additional value of £30m to be gained from further research to determine whether ‘IUT’ is cost-effective compared to ‘no IUT’ in investigating women with SUI and stress-predominant MUI prior to surgical treatment. The financial (£1.8m) and opportunity (£8.9m) costs of further research were subtracted from the EVSI to estimate the ENG (£19m). The optimal sample size was estimated as 91 women randomised to each arm; after inflation for non-responses to the SF-12 questionnaires, the optimal sample size was 404 women in total. If achieved, in a future definitive trial, this sample size would maximise ENG and should result in a definitive conclusion on the cost-effectiveness of IUT.

#### Sensitivity analysis

Sensitivity analysis was performed to determine the impact on ENG and optimal sample size when we imputed the unadjusted EQ-5D-3L cost-effectiveness results. The NMB based on the EQ-5D-3L unadjusted cost-effectiveness results was £216 (95% CI − £197, £269) in favour of ‘no IUT’; hence, the assumption for the EVSI was to estimate the additional value of future research to determine whether ‘no IUT’ was cost-effective compared to ‘IUT’. It is important to note that this difference in the assumption was driven by the increased QALYs experienced by ‘no IUT’.

The results estimated from the EVSI model, presented in Additional file 5, suggest there is additional value of £61m to be gained from further research. The optimal sample size was estimated as 103 women randomised to each arm; after inflation for non-responses to the EQ-5D-3L questionnaires, the optimal sample size was 416 women (note the SF-12 and EQ-5D-3L had different response rates and were inflated accordingly). The sample size required to maximise society’s ENG was relatively consistent when we imputed the EQ-5D-3L and SF-12 results (416 women vs. 404 women).

## Discussion

It must be emphasised that INVESTIGATE-I was planned as a mixed methods feasibility study with the primary objective of informing the decision whether or not to proceed to a definitive randomised comparative trial and whether or not any refinements to the design or conduct of such a trial are warranted; hence, the pilot trial within the INVESTIGATE-I package was not powered to provide definitive answers to either clinical or economic outcomes. It follows that the VoI analysis builds upon an inherently ‘underpowered economic evaluation’, with the aim of establishing how large an adequately powered definitive study would need to be.

Despite its limited statistical power, the pilot trial undertaken within the INVESTIGATE-I package remains one of the largest studies undertaken addressing the clinical utility of IUT in SUI and stress-predominant MUI, and the largest to consider cost-effectiveness [15]. During the planning of INVESTIGATE-I, two other trials were identified as ongoing, and both have since been published [5, 8]. Although the protocols were rather different, both employed a non-inferiority design and reported no benefit from IUT. Both of these earlier studies included women with SUI or stress-predominant MUI, but in contrast to INVESTIGATE-I, both also required that urine leakage on stress should be clinically demonstrated. It has long been recognised that patients with predominant symptoms of SUI who also have clinically demonstrable stress leakage are very likely (97%) to have urodynamic stress incontinence on invasive testing [35], and some clinical guidelines have followed this evidence base [36, 37]. Our intention in the feasibility study INVESTIGATE-I was to broaden the inclusion criteria, to include those in whom stress leakage was not necessarily clinically demonstrated, so that we might potentially influence policy and practice for a greater number of women following a future definitive trial.

In a model-based economic evaluation in women with overactive bladder and/or urge-predominant MUI, it was found that IUT was cost-effective only in women with urge-predominant MUI and that women with overactive bladder symptoms should receive treatment on the basis of the clinical diagnosis [38, 39]. The question of how much different is the cost-effectiveness of IUT in women with SUI only compared to women with stress-predominant MUI is therefore pertinent. Although we have not undertaken such a sub-group analysis in this feasibility study, it could usefully be incorporated into a future definitive trial.

It has been argued on the basis of systematic review and meta-analysis that comprehensive clinical assessment is a sufficient investigation in the diagnosis of SUI or stress-predominant MUI without voiding difficulties, as long as the women undergo careful office evaluation [40]. In contrast, Serati et al. found that, even in women with a preliminary categorisation of ‘uncomplicated SUI’, the additional information provided from invasive investigation can alter treatment [41]; they did not however provide data on the outcomes from treatment. In INVESTIGATE-I, there was a 20% reduction (*p* < 0.01) in surgical treatment in the ‘IUT’ arm suggesting that IUT provided additional information which influenced treatment decisions. In addition, only a small number of women report pure symptoms of SUI and hence satisfy current NICE criteria for undergoing clinical assessment only [42]. Whilst we would therefore concur that IUT influences treatment, the issue of how far it affects treatment outcome remains unresolved.

In previous work, it has been implied that there would be cost-savings associated with omitting IUT from the diagnostic pathway of SUI and stress-predominant MUI; however, there is no evidence to support this conclusion as the studies included in the meta-analysis provided no information on cost-effectiveness [40]. Our results do not support this assumption as cost-savings (mean diff − £138; *p* = 0.07) were associated with IUT. The bootstrapped 95% CI (− £258, £12) does include 0 but the upper end of the CI, which would favour ‘no IUT’, is close to zero, and possibly of no economic significance as less than 1% of the bootstrapped iterations would support there being cost-savings from ‘no IUT’. Perhaps the more appropriate interpretation of these results is that for one in five women there is a potential cost-saving of £1244 from the IUT strategy as a result of avoiding surgical treatment. These cost-savings would be reduced by the costs of the investigation (IUT) itself and subsequent non-surgical treatments prescribed as a consequence. However, given that non-surgical treatments are relatively low cost compared to surgery, it is anticipated that cost-savings would still result. It must be emphasised that INVESTIGATE-I was a pilot study and the economic analyses were not powered to determine whether these cost-savings were statistically significant. Nevertheless, possible additional costs are small and of questionable economic significance.

Both unadjusted and adjusted analyses were performed as part of the economic analysis. The importance of using SUR to adjust for potential imbalances in the trial data, due to individual characteristics, which could influence costs and effects is widely recognised [29]. The implications of not adjusting for these imbalances could result in the differences in outcomes being overestimated and hence could result in policy-makers being misinformed. In both of our analyses, the estimated incremental cost difference remained consistent; however, the unadjusted analysis overestimated the incremental difference in QALYs, particularly the EQ-5D-3L results. This difference in QALYs has affected the ICER in that ‘no IUT’ would be considered cost-effective in the unadjusted analysis but not the adjusted analysis at current willingness-to-pay thresholds [31]. For a future definitive study, we would recommend the use of both analyses to estimate costs and effects as this would reduce any potential uncertainty around the probability of IUT being considered cost-effective.

It is acknowledged that there is a large amount of missing data in these analyses, with less than 50% of patients returning completed questionnaires at both baseline and 6 months, 48% had QALYs estimated from the EQ-5D-3L and 45% from the SF-12. Although the uncertainty caused by the dropout rate is incorporated into the analyses, the need for caution in interpretation is clear, particularly since somewhat more data were missing from the intervention than control arms [16]. Potential solutions to increase the response rates are presented elsewhere [16].

The difference in the utility scores estimated from the EQ-5D-3L and SF-12 is not surprising as they both capture different dimensions to estimate quality of life and have different recall periods; the EQ-5D-3L asks participants to measure their health today and the SF-12 asks participants to recall their health over the last 4 weeks [43]. In the case of INVESTIGATE-I, the SF-12 is arguably more sensitive to changes in UI and has been shown to be more responsive than the EQ-5D for capturing changes in less chronic conditions [44]. That being said, going forward, the EQ-5D is recommended by NICE [31] and the introduction of the EQ-5D-5L has increased the sensitivity of the questionnaire [45]. In addition, the response rate to the EQ-5D tends to be higher [43], which was also evident from this study; hence, the EQ-5D-5L should be considered as the preferred economic outcome measure in a future study.

To our knowledge, this is the first full economic evaluation comparing IUT with basic clinical assessment to basic clinical assessment alone in the investigation of SUI and stress-predominant MUI in women prior to surgical treatment, albeit in the context of a feasibility study. As previously mentioned, the advantage of including IUT in the investigation of SUI and stress-predominant MUI in women prior to surgical treatment is that women would receive treatment tailored to their diagnosis and potentially avoid unnecessary surgery [41]. This could generate cost-savings as non-surgical treatments are relatively low cost compared to surgical treatment. Although performing IUT could change the diagnosis or treatment, it does not necessarily mean that the woman’s quality of life improves (this is supported by the QALY results and the results of the Value of Urodynamic Evaluation (ValUE) trial) [5]. Longer-term data are needed to identify whether women experience any changes in outcomes over time and whether the cost-savings associated with IUT are maintained in the long term. If a woman who has avoided surgical treatment on the basis of IUT continues with non-surgical treatment in the long term, then cost-savings may be maintained. If, on the other hand, she does not benefit from conservatism, and later goes on to have surgical treatment, then clearly no savings will have been made, and indeed additional costs will accrue. Within INVESTIGATE-I, no patients went on to have surgery after initial non-surgical management, perhaps because of the short time horizon of the pilot; in a definitive trial, we would recommend that a minimum 12 month follow-up be considered.

Norton et al. performed a cost simulation on the results of the ValUE trial as a secondary analysis to determine the costs associated with urodynamic studies (UDS) [46]. They concluded that millions of dollars could be saved in the USA by omitting preoperative UDS in women with uncomplicated stress-predominant urinary incontinence prior to surgical treatment [46]. However, given their inclusion criteria, this is unsurprising as the majority of women who received UDS underwent surgical treatment (94.6%) [5]. Their conclusions should not be generalised outside of their patient group and the USA, hence are not applicable to our population.

Where our data are useful is in the judgement of whether further research to address the uncertainty in the results would be valuable. The VoI analysis suggests that there is added value (at least £19 million for the UK alone) to be gained from additional research. It is reassuring that despite the lack of statistical significance surrounding our results, a conclusion can be drawn that there are potential cost-savings associated with IUT and additional value to be gained from further research. The primary clinical outcome used in the INVESTIGATE-I pilot trial was the International Consultation on Incontinence Modular Questionnaire (ICIQ) Female Lower Urinary Tract Symptoms (ICIQ-FLUTS) total score. There is consistency in the sample size calculations estimated from the standard statistical criteria looking for a minimum clinical difference of 3 points in ICIQ-FLUTS score (*n* = 410) [16] and the EVSI results for both the SF-12 (*n* = 404) and EQ-5D-3L (*n* = 416). Using all three estimations to determine the sample size of a future definitive trial means the trial will be powered to determine both the clinical and economic effectiveness of IUT. This combination of approaches is rarely performed and is consistent with recent recommendations on sample size determination [47].

As with any sample size calculation, there are uncertainties [48]. In the case of sample sizes based on the EVSI results, one uncertainty is around the methodological approach to estimate cost-effectiveness. Arguably, the better method is to estimate cost-effectiveness based on the SUR approach and hence base estimates of the EVSI on these results. This was not possible as not all of the required data were available. Had it been possible, we would expect the estimated sample size to be slightly higher.

Although it is an overused conclusion in clinical research and reviews, we can say with some certainty that there is additional value to be gained from future research to evaluate the clinical and cost-effectiveness of IUT in the investigation of SUI and stress-predominant MUI in women prior to surgical treatment. Future studies should consider expanding the time horizon beyond 6 months to determine the long-term benefits associated with surgical and non-surgical treatment options, incorporate the woman’s perspective in the economic evaluation and, if using similar outcome measures, should consider using the sample size estimates determined in the current VoI analysis and previously published conventional power calculations [16]. The combination of our economic results with the economic results of a future definitive trial should result in sufficient evidence to inform NICE or other policy making organisations internationally in making recommendations on the value of IUT in the diagnostic pathway of SUI and stress-predominant MUI [49].

## Conclusion

This is the first full economic evaluation of IUT in diagnosing SUI or stress-predominant MUI in women whom stress leakage was not necessarily clinically demonstrated, albeit in the context of a feasibility study. Our findings suggest that IUT may be cost-saving compared to basic clinical assessment and non-invasive tests in the investigation of SUI and stress-predominant MUI prior to surgical treatment, assessed over the short term. There is additional net value to be gained from further research in this area and the question should be addressed by a trial that is both appropriately powered to detect clinical and economic differences between IUT and basic clinical assessment, and extended over a longer time scale. A sample size of 404–416 randomised women should be capable of providing definitive answers to these questions.

## Additional files

Additional file 1: Unit costs. (DOCX 17kb)

Additional file 2: Average resource use per randomised arm. (DOCX 15kb)

Additional file 3: Average utility values per randomised arm. (DOCX 13kb)

Additional file 4: EVSI model input data (using the SF-12 analysis). (DOCX 15kb)

Additional file 5: Expected value of sampling information derived from EQ-5D-3L data. (JPG 67kb)

## Abbreviations

ENG: Expected net gain
EVSI: Expected value of sampling information
GP: General practitioner
ICER: Incremental cost-effectiveness ratio
ICIQ: International Consultation on Incontinence Modular Questionnaire
ICIQ-FLUTS: ICIQ - Female Lower Urinary Tract Symptoms
INVESTIGATE-I (*study acronym*): INVasive Evaluation before Surgical Treatment of Incontinence Gives Added Therapeutic Effect?
IUT: Invasive urodynamic testing
MUI: Mixed urinary incontinence
NHS: National Health Service
NICE: National Institute for Health and Care Excellence
NIHR-HTA: National Institute for Health Research – Health Technology Assessment
NMB: Net monetary benefit
QALY: Quality-adjusted life year
SUI: Stress urinary incontinence
SUR: Seemingly unrelated regression
UDS: Urodynamic studies
UI: Urinary incontinence
ValUE: Value of Urodynamic Evaluation
VoI: Value of information
WTP: Willingness-to-pay
