# Supplementary material for: Invasive urodynamic testing prior to surgical treatment for stress urinary incontinence in women: cost-effectiveness and value of information analyses in the context of a mixed methods feasibility study
Source: Pilot Feasibility Stud. 2018 Mar 23;4:67. doi: 10.1186/s40814-018-0255-y (PMC5865344; doi:10.1186/s40814-018-0255-y)
Supplement: Supplementary file 1 — Unit costs. (DOCX 17kb) [file 40814_2018_255_MOESM1_ESM.docx]

**Additional file 1** Unit costs

| **Resource use** | **Cost per unit (£)** | **Source/Note** |
| --- | --- | --- |
| **Cost of the intervention (IUT)** |  |  |
| Cost of consumable items  - Pump set, Bladder irrigation litre bags, Pressure domes x 2, 10ml syringes x 2, dual lumen filling and vesical pressure lines and abdominal pressure line: combined pack, catheter pack, Intillagel (anaesthetic lubricating gel) syringe, KY lubricating gel – sachet, Normasol sachet, Tissues, Yellow bags x 1, Gloves disposable x 4, MSU specimen bottle x 1, Urinalysis Mulristix 8SG) | 27.20 | Micro costing |
| Cost of capital resources^#^ | 11.04 | Micro costing |
| Cost per minute of consulting room | 0.45 | Micro costing |
| Cost per minute of staff – grade 3 | 0.19 | Pay scales 2015 |
| Cost per minute of staff – grade 5 | 0.26 | Pay scales 2015 |
| Cost per minute of staff – grade 6 | 0.32 | Pay scales 2015 |
| Cost per minute of staff – grade 8 | 0.47 | Pay scales 2015 |
| Cost per minute of staff – Consultant | 1.70 | PSSRU 2014 |
| Cost per minute of staff – SpR/SST | 0.67 | PSSRU 2014 |

^#^ Urodynamic equipment costs per participant assumed a ten-year life span, an annual usage of 550 investigations and a 3.5% discount rate.

**Table S1** Unit costs cont.

| **Resource use** | **Cost per unit (£)** | **Source/Note** |
| --- | --- | --- |
| **Cost of surgery** |  |  |
| Cost of TVT surgery | 1244 | NHS ref costs 2013-14 |
| Cost of admission – day | 288 | NHS ref costs 2013-14 – urinary incontinence and other urinary problems without CC. |
| Cost of admission – night | 370 | NHS ref costs 2013-14 |
| **Follow-up – Secondary care** |  |  |
| Inpatient visits | 370 | NHS ref costs 2013-14 |
| Outpatient visits | 99 | NHS ref costs 2013-14 - Urology dept. |
| **Follow-up – Primary care** |  |  |
| GP practice visits | 38 | PSSRU 2014 |
| GP home visits | 96.50 | PSSRU 2014 |
| GP phone consultation | 23 | PSSRU 2014 |
| Practice nurse visit | 11.37 | PSSRU 2014 |
| Continence nurse visit | 22 | PSSRU 2014 – nurse advanced |
| Physio visit | 16 | PSSRU 2014 |
| Prescription | 8.60 | PSSRU 2014 |

**Table S1** Unit costs cont.

| **Resource use** | **Cost per unit (£)** | **Source/Note** |
| --- | --- | --- |
| **Travel** | **Travel** | **Travel** |
| Hospital car | 10.06 | ISD 2014 - Table R910 |
| Ambulance | 99 | NHS ref costs 2013-14 |
| **Cost of other treatments** |  |  |
| Bladder retraining* | 378 | NHS ref costs 2013-14 |
| PFMT** | 112 | PSSRU 2014 |
| Alternative behaviour modification*** | 22 | NHS ref costs 2013-14 |
| Watchful waiting (containment products) | 50 | Imamura *et al* HTA report (2010) inflated to 2015 values^[^[^21^](#_ENREF_21)^,^ [^22^](#_ENREF_22)^]^ |
| **Antimuscanaric drugs (six-month dosage)**  Solifenacin 5mg | 165 | medicinescomplete.com 2015 |
| Solifenacin 10mg | 215 | medicinescomplete.com 2015 |
| Oxybutynin 5mg | 28 | medicinescomplete.com 2015 |

*Based on 1 new gynaecological appointment and 2 follow up appointments; **Based on a 1 hour physio appointment and 5 half an hour follow up appointments; ***Based on 15 minute consult with continence nurse
