# Supplementary material for: Invasive urodynamic testing prior to surgical treatment for stress urinary incontinence in women: cost-effectiveness and value of information analyses in the context of a mixed methods feasibility study
Source: Pilot Feasibility Stud. 2018 Mar 23;4:67. doi: 10.1186/s40814-018-0255-y (PMC5865344; doi:10.1186/s40814-018-0255-y)
Supplement: Supplementary file 2 — Average resource use per randomised arm. (DOCX 15kb) [file 40814_2018_255_MOESM2_ESM.docx]

| **Additional file** **2** Average resource use per randomised arm | | | | | | | | | |
| --- | --- | --- | --- | --- | --- | --- | --- | --- | --- |
| **Health care resource** |  | | **‘no IUT’ (N=108)** | | | **‘IUT’ (N=110)** | | |  |
|  | **Units** | **N** | | **Mean (SD)** | **N** | | **Mean (SD)** | **P-value** | |
| Duration of IUT | Minutes | - | | - | 89 | | 40.0 (11.0) |  | |
| Number of women undergoing surgical treatment | Surgeries | 101 | | - | 81 | | - | **<0.01*** | |
| Number of women undergoing surgical treatment as a day case | Day cases | 100 | | - | 80 | | - | 0.88 | |
| Length of admission for surgery if as an inpatient | Nights | 1 | | 1.0 (0.0) | 1 | | 1.0 (0.0) | 0.88 | |
| Number of women who completed the PCQ part A | Questionnaires | 66 | | - | 50 | | - |  | |
| Number of GP practice visits | Visits | 10^1^ | | 2.5 (1.6) | 10 | | 2.4 (1.8) | 0.90 | |
| Number of GP home visits | Visits | 10 | | 0.0 (0.0) | 9 | | 0.0 (0.0) | - | |
| Number of GP Phone consults | Visits | 10 | | 1.1 (2.0) | 9 | | 1.4 (3.2) | 0.78 | |
| Number of Practice nurse visits | Visits | 6 | | 2.0 (0.9) | 3 | | 2.7 (1.2) | 0.37 | |
| Number of Continence nurse visits | Visits | 3 | | 1.3 (0.6) | 4 | | 2.0 (1.4) | 0.48 | |
| Number of Physio visits | Visits | 2 | | 1.5 (0.7) | 3 | | 5.3 (4.0) | 0.30 | |
| Number of Outpatient visits | Visits | 14 | | 2.1 (0.9) | 14 | | 1.9 (0.7) | 0.46 | |
| Number of Inpatient visits | Nights | 8 | | 0.5 (0.9) | 6 | | 1 (1.5) | 0.29 | |
| Number of Prescriptions | Scripts | 3 | | 1.7 (1.1) | 8 | | 3.5 (2.7) | 0.30 | |

N = number of participants in each study arm who had information available; SD = standard deviation; *Statistically significant; ^1^Of the 66 women in the ‘no IUT’ arm who completed the PCQ part A, 10 reported having a GP practice consultation; the average number of GP consultations among those 10 participants was 2.5.
