# Supplementary material for: Invasive urodynamic testing prior to surgical treatment for stress urinary incontinence in women: cost-effectiveness and value of information analyses in the context of a mixed methods feasibility study
Source: Pilot Feasibility Stud. 2018 Mar 23;4:67. doi: 10.1186/s40814-018-0255-y (PMC5865344; doi:10.1186/s40814-018-0255-y)
Supplement: Supplementary file 3 — Average utility values per randomised arm. (DOCX 13kb) [file 40814_2018_255_MOESM3_ESM.docx]

**Additional file 3** Average utility values per randomised arm

| **Utility measures** |  | **‘no IUT’ (N=108)** | | **‘IUT’ (N=110)** | | |  |
| --- | --- | --- | --- | --- | --- | --- | --- |
|  |  | **N** | **Mean (SD)** | **N** | **Mean (SD)** | **P-value** |  |
| Baseline (EQ-5D 3L) |  | 85 | 0.86 (0.19) | 75 | 0.84 (0.24) | 0.32 | |
| Six-months (EQ-5D 3L) |  | 65 | 0.91 (0.18) | 49 | 0.88 (0.27) | 0.33 | |
| **QALYs (EQ-5D 3L)** |  | 60 | 0.45 (0.08) | 45 | 0.44 (0.11) | 0.24 | |
| Baseline (SF-12) |  | 81 | 0.75 (0.13) | 69 | 0.75 (0.13) | 0.79 | |
| Six-months (SF-12) |  | 65 | 0.78 (0.14) | 47 | 0.79 (0.16) | 0.89 | |
| **QALYs (SF-12)** |  | 59 | 0.38 (0.06) | 39 | 0.39 (0.7) | 0.40 | |

N = number of participants in each study arm who had information available; SD = standard deviation; QALY = quality-adjusted life years; *statistically significant at the 5% level
