# Supplementary material for: Invasive urodynamic testing prior to surgical treatment for stress urinary incontinence in women: cost-effectiveness and value of information analyses in the context of a mixed methods feasibility study
Source: Pilot Feasibility Stud. 2018 Mar 23;4:67. doi: 10.1186/s40814-018-0255-y (PMC5865344; doi:10.1186/s40814-018-0255-y)
Supplement: Supplementary file 4 — EVSI model input data (using the SF-12 analysis). (DOCX 15kb) [file 40814_2018_255_MOESM4_ESM.docx]

**Additional file 4** EVSI model input data (using the SF-12 analysis)

| **t** | **Description** | **Value** | **Source/Justification** |
| --- | --- | --- | --- |
| nT | Number of people in Tx**^A^** arm | 110 | Trial data |
| Mean E T | Mean QALY Tx | 0.385 | Trial data |
| Var E T | Variance QALY Tx | 0.003 | Trial data |
| Mean C T | Mean cost Tx | 1351 | Trial data |
| Var C T | Variance cost Tx | 460,843 | Trial data |
| Cov EC T | Covariance (QALY / Cost) Tx | 20.46 | Trial data |
| nS | Number of people in Cx**^B^** arm | 108 | Trial data |
| Mean E S | Mean QALY Cx | 0.4047 | Trial data |
| Var E S | Variance QALY Cx | 0.009 | Trial data |
| Mean C S | Mean cost Cx | 1489 | Trial data |
| Var C S | Variance cost Cx | 180,274 | Trial data |
| Cov EC S | Covariance (QALY / Cost) Cx | 17.10 | Trial data |
| h | Time Horizon (life expectancy of intervention) | 10 years | Author’s assumption – IUT will be available for 10 years reflecting the possible future changes in technology, price and information. |

**Table S4** EVSI model input data (using the SF-12 analysis) cont.

| K | Annual Incidence (of condition under study) | 12,000 | Annual number of women undergoing surgery^*^ |
| --- | --- | --- | --- |
| A | Annual Accrual (rate into trial) | 127 (148) | Trial data – from recruitment start date (from first patient recruited) |
| Tau | Follow-up/Analysis (duration from last recruited patient to results being available). | 1 | Trial data **-** 6 months follow-up, followed by 6 months to analyse the data and for the results to become public knowledge. |
| Cf | Fixed Cost (of running trial) | £1,477,202 | Total research costs requested for definitive trial (not including NHS support and treatment costs) |
| Cv | Variable Cost (of recruiting 1 patient) | £1,602 | Based on the cost of recruiting 1 additional patient |
| CA | Cost of Adoption | 0 | Assume £0 cost of adoption. |
| Lambda (λ) | Threshold value WTP**^C^** for a QALY | £20,000 | As per NICE reference case |

* Sourced from Hospital Episode Statistics (2014). Department of Health: [www.hesonline.nhs.uk](http://www.hesonline.nhs.uk).
