# Supplementary figures and images for: Invasive urodynamic testing prior to surgical treatment for stress urinary incontinence in women: cost-effectiveness and value of information analyses in the context of a mixed methods feasibility study
Source: Pilot Feasibility Stud. 2018 Mar 23;4:67. doi: 10.1186/s40814-018-0255-y (PMC5865344; doi:10.1186/s40814-018-0255-y)

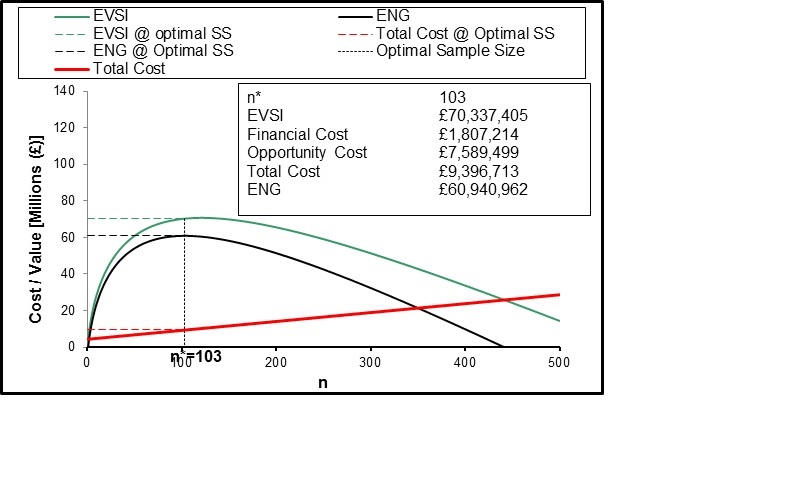

Supplement: Supplementary file 5 — Expected value of sampling information derived from EQ-5D-3L data. (JPG 67kb) [file 40814_2018_255_MOESM5_ESM.jpg]
